# O^6^-Methylguanine-DNA Methyltransferase (MGMT) mRNA Expression Predicts Outcome in Malignant Glioma Independent of *MGMT* Promoter Methylation

**DOI:** 10.1371/journal.pone.0017156

**Published:** 2011-02-18

**Authors:** Simone Kreth, Niklas Thon, Sabina Eigenbrod, Juergen Lutz, Carola Ledderose, Rupert Egensperger, Joerg C. Tonn, Hans A. Kretzschmar, Ludwig C. Hinske, Friedrich W. Kreth

**Affiliations:** 1 Department of Anaesthesiology, Ludwig Maximilians University, Munich, Germany; 2 Department of Neurosurgery, Ludwig Maximilians University, Munich, Germany; 3 Center for Neuropathology and Prion Research, Ludwig Maximilians University, Munich, Germany; 4 Department of Radiology, Ludwig Maximilians University, Munich, Germany; 5 Department of Anaesthesiology, University Medical Center Mannheim, Mannheim, Germany; Dana-Farber Cancer Institute, United States of America

## Abstract

**Background:**

We analyzed prospectively whether MGMT (O^6^-methylguanine-DNA methyltransferase) mRNA expression gains prognostic/predictive impact independent of *MGMT* promoter methylation in malignant glioma patients undergoing radiotherapy with concomitant and adjuvant temozolomide or temozolomide alone. As DNA-methyltransferases (DNMTs) are the enzymes responsible for setting up and maintaining DNA methylation patterns in eukaryotic cells, we analyzed further, whether *MGMT* promoter methylation is associated with upregulation of DNMT expression.

**Methodology/Principal Findings:**

Adult patients with a histologically proven malignant astrocytoma (glioblastoma: N = 53, anaplastic astrocytoma: N = 10) were included. *MGMT* promoter methylation was determined by methylation-specific PCR (MSP) and sequencing analysis. Expression of MGMT and DNMTs mRNA were analysed by real-time qPCR. Prognostic factors were obtained from proportional hazards models. Correlation between MGMT mRNA expression and *MGMT* methylation status was validated using data from the Cancer Genome Atlas (TCGA) database (N = 229 glioblastomas). Low MGMT mRNA expression was strongly predictive for prolonged time to progression, treatment response, and length of survival in univariate and multivariate models (p<0.0001); the degree of MGMT mRNA expression was highly correlated with the *MGMT* promoter methylation status (p<0.0001); however, discordant findings were seen in 12 glioblastoma patients: Patients with methylated tumors with high MGMT mRNA expression (N = 6) did significantly worse than those with low transcriptional activity (p<0.01). Conversely, unmethylated tumors with low MGMT mRNA expression (N = 6) did better than their counterparts. A nearly identical frequency of concordant and discordant findings was obtained by analyzing the TCGA database (p<0.0001). Expression of DNMT1 and DNMT3b was strongly upregulated in tumor tissue, but not correlated with *MGMT* promoter methylation and MGMT mRNA expression.

**Conclusions/Significance:**

MGMT mRNA expression plays a direct role for mediating tumor sensitivity to alkylating agents. Discordant findings indicate methylation-independent pathways of MGMT expression regulation. DNMT1 and DNMT3b are likely to be involved in CGI methylation. However, their exact role yet has to be defined.

## Introduction

World Health Organisation (WHO) Grade III anaplastic astrocytoma (AA) and WHO grade IV glioblastoma (GBM) are rapidly progressive and resistant to therapy. Thus, malignant glioma patients suffer the devastating effects of an incurable disease with short survival times after diagnosis. More recently, some progress has been achieved in the treatment of these tumors: Prospective randomized studies of the European Organisation for Research and Treatment of Cancer (EORTC) and the National Cancer Institute of Canada (NCIC) trial have shown that the addition of the alkylating agent temozolomide (TMZ) to radiotherapy (XRT) for newly diagnosed GBM resulted in significant prolongation of both time to progression and overall survival. As a result, median survival which has been estimated in the range one year for GBM and three years for AA [1], [2], [3] has slightly been increased. Moreover, molecular markers have been identified, which determine the course of the disease. An important biomarker is the methylation status of the O^6^-methylguanine-DNA methyltransferase (*MGMT*) gene promoter. Epigenetic silencing of the *MGMT* gene has been identified as a strong and independent predictive factor of treatment response for both GBM- and AA-patients undergoing chemotherapy with alkylating agents [4], [5]. Correlations between promoter methylation and favorable treatment response after chemotherapy with TMZ or other alkylating agents are explained by the assumption that DNA methylation of a cysteine-phosphate-guanine (CpG) island (CGI) within the *MGMT* promoter directly leads to a repression of M*GMT* transcriptional activity and MGMT protein expression [6]; determination of the promoter methylation status may thus serve as a “chemosensitivity sensor” in glioma patients. This hypothesis, however, which implies that MGMT promoter methylation status, MGMT expression data and outcome measurements are strongly correlated with each other, has not unequivocally been supported: Studies evaluating MGMT expression by immunohistochemistry (IHC), for example, mostly failed to detect correlations between MGMT expression, *MGMT* methylation status and outcome measurements [7]–[9]. One more recently published study on transcriptional activity in glioblastomas questions mechanisms of “direct” transcriptional repression by *MGMT* promoter methylation for a considerable number of tumors: Even though overall a strong correlation between *MGMT* promoter methylation and the degree of MGMT mRNA expression was found [10], discordant findings were seen in at least 15% of the investigated tumors, i.e. unmethylated (methylated) tumors expressed low (high) levels of MGMT mRNA. Unfortunately, this study did not provide any correlative data between MGMT mRNA expression and clinical outcome to further support the view of a sometimes “broken link” between *MGMT* promoter methylation and mRNA expression.

The objective of the present study was to prospectively investigate the predictive impact of *MGMT* gene expression under consideration of its correlation with the *MGMT* promoter methylation status in malignant glioma patients undergoing XRT and/or TMZ treatment. As aberrant DNA (cytosine-5)-methyltransferase (DNMT) expression has been observed in several tumor tissues [11]–[13] which might – at least in part – explain epigenetic silencing of selected genes by promoter methylation, we additionally estimated the expression of DNMTs in tumor tissue as compared to normal brain, its prognostic/predictive relevance in malignant glioma, and its correlation with both the *MGMT* promoter methylation status and MGMT mRNA expression levels.

## Methods

### Study design

Adult patients were eligible if they had i) a supratentorial GBM or AA with histology being proven by stereotactic biopsy or open tumor resection (May 2007 to March 2009), no prior history of surgery, XRT, and/or chemotherapy, and a Karnofsky performance score (KPS) ≥60 [14]. All patients gave written informed consent, and the prospective study protocol was reviewed and approved by the institutional review board of the Ludwig Maximilians University, Munich, Germany (AZ 216/14). Indication for either surgical procedure was dependent on tumor size and location, mass effects of the tumor, patients' KPS and/or significant co-morbidity. In case of moderate space occupying effects of the tumor, a highly eloquent tumor location, and/or significant co-morbidity stereotactic biopsy was preferred. Histopathological diagnosis, determination of the *MGMT* promoter methylation status and *MGMT* transcriptional activity were obtained within 8–12 working days after surgery. Within 3 weeks upon histopathological diagnosis, patients with GBM were assigned to receive XRT plus concomitant and adjuvant TMZ (XRT/TMZ→TMZ). Treatment parameters were as follows: XRT (60 Gy in 30 fractions)/TMZ (daily dose of 75 mg/m^2^)→TMZ (150 to 200 mg/m^2^ per day for 5 days of every 28-day cycle). In case of long term compliance, TMZ was continued (at the same dose) until tumor progression occurred, which indicated a difference to the EORTC treatment protocol [2]. Patients with the diagnosis of an AA were treated according the EORTC protocol [2] in case of an extraaordinarily high Ki67 labelling index (>20%), otherwise primary chemotherapy with TMZ was initiated and XRT was withheld [5]. At baseline evaluation, within 72 h after cytoreductive surgery, 4–6 weeks after XRT/TMZ and every 3 cycles during TMZ maintenance therapy, neuroradiologic examinations were performed. Early treatment response was evaluated after the completion of 3 TMZ cycles or earlier in case of clinical deterioration. Magnetic resonance image (MRI) interpretation was independently done according to the Macdonald criteria [15] by an experienced neuroradiologist (JL), who was blinded for the *MGMT* methylation status and transcriptional activity as well as for the follow up data of the patients. Tumor progression had to be confirmed by further clinical and neuroradiological follow up to exclude any bias by pseudoprogression [16]. Haematology was performed weekly. Adverse events were defined according to the National Cancer Institute (NCI) Common Toxicity Criteria, version 3.0. The minimum follow up after inclusion of the last patient had to be 6 months.

### Histopathology

For histopathological evaluation, tissue samples harvested from either cytoreductive surgery or biopsy procedures were fixed with 4% buffered formalin, paraffin embedded and subjected to routine stainings (Hematoxylin and Eosin, Elastica van Gieson, Periodic acid-Schiff) and IHC with antibodies against human GFAP (monoclonal mouse, clone 6F2, Dako, Glostrup, Denmark) and anti-MAP2 (clone HM-2, Sigma, Saint Louis, Missouri, USA). Proliferation activity was determined using anti-human Ki67 antigen (mouse monoclonal, clone MIB-1, Dako, Glostrup, Denmark). The histological diagnosis of all tissue specimens was made according to WHO criteria [17].

### Tissue sampling

Glioma tissue samples for molecular genetic analysis were obtained from fluorescence-guided open tumor resections [18] or serial stereotactic biopsy procedures [19], [20]. Molecular genetic evaluation of tissue samples obtained from open tumor resection was exclusively done in tissue samples in the direct vicinity of samples showing solid tumor tissue. In case of biopsy, co-registration of computerized tomography (CT), and MRI (including T1- and T2-weighted sequences) served for 3D visualization (i-plan stereotaxy®, BrainLAB®, Feldkirchen, Germany) of the tumor and the simulation of the best biopsy trajectory representative of the solid tumor. Serial biopsies were taken in one-millimeter steps exactly along the chosen trajectory. Using micro forceps the maximum amount of tissue per biopsy specimen was 1 mm^3^. The number of specimens taken was in the range of 10–18 samples per tumor. The tissue sampling procedure was guided by intra-operative smear preparations, which were routinely performed by the attending neuropathologist: Only tumor probes next (i.e. 1 mm distance) to smear preparations exclusively showing solid vital tumor tissue were selected for molecular genetic analysis; a corresponding sample (level +1 mm), which was taken for paraffin embedding and histopathological examination using standard protocols [20], also had to show solid vital tumor tissue. The described biopsy technique was chosen to minimize the risk of tissue contamination (e.g. by non-neoplastic or necrotic tissue) and more importantly, to recognize contamination, if it occurs. For the detection of potential heterogeneity of *MGMT* promoter methylation and MGMT mRNA expression throughout the solid tumor space, biopsy specimens selected for molecular-genetic analyses were harvested from at least two different sites along the chosen trajectory of each tumor in the biopsy group. Normal brain (from 9 patients) was obtained from epilepsy surgery. One additional normal brain sample mRNA was purchased from Ambion (Ambion, Austin, USA).

### Combined RNA and DNA Isolation

A sequential purification procedure for both DNA and RNA was performed as being published before [19]. Briefly, RNA was isolated using RNAqueous® Micro Kit (Ambion®, Austin, TX, USA), and in a second step DNA was extracted using the QIAmp® DNA Micro Kit (Qiagen®, Hilden, Germany) from the first flow-through of RNA isolation following lysis of the sample. The quantity and purity of the obtained nucleic acids was assessed using the NanoDrop® ND-1000 spectrophotometer (NanoDrop®, Wilmington, DE, USA).

### Methylation-specific PCR (and sequencing analysis)

Exclusively histopathologically verified solid viable tumor tissue was used for determination of *MGMT* promoter methylation and measurements of transcriptional activity. Isolation of nucleic acids, bisulfite modification of DNA, methylation-specific PCR (MSP) and sequencing analyses were performed as being published in detail before [20]. In brief, DNA isolation from tumor specimens was performed using commercially available isolation kits followed by purification and bisulfite-modification of DNA [21]. For MSP 2 pairs of primers, each specific for either the methylated or the unmethylated *MGMT* promoter region, were used as described by Esteller and collegues [22]. Unmethylated versus methylated tumors were defined as described by Grasbon-Frodl et al. [20].

### Linear amplification and reverse transcription of RNA

20–50 ng of purified RNA of all samples were amplified using the TargetAmp-Kit (Epicentre, Madison, Wisconsin, USA) according manufacturer's recommendations in order to obtain RNA amounts suitable for gene expression analyses [23]. The resulting amplification factors were between 500 and 2500. Hereafter, equal amounts of the different samples of amplified RNA (1000 ng) were transcribed into cDNA. The RT reaction was carried out using random primers and Superscript III reverse transcriptase (Invitrogen, Carlsbad, USA), as per manufacturer's instructions.

### Real-time PCR

Real-time qPCR was performed in triplicates with the Light Cycler 480 instrument (Roche Diagnostics, Mannheim, Germany) using Roche's qPCR Mastermix and highly specific Universal ProbeLibrary assays (Roche Diagnostics). The following primers were used: *MGMT:* 5′- GTGATTTCTTACCAGCAATTAGCA-3′ (forward primer), 5′- CTGCTGCAGACCACTCTGTG-3′ (reverse primer); Probe: Universal ProbeLibrary probe: #52. *TBP*: 5′- GAACATCATGGATCAGAACAACA-3′ (forward primer), 5′- ATAGGGATTCCGGGAGTCAT-3′ (reverse primer); Probe: Universal ProbeLibrary probe: # 87. *SDHA:* 5′- GAGGCAGGGTTTAATACAGCA-3′ (forward primer), 5′- CCAGTTGTCCTCCTCCATGT-3′ (reverse primer); Probe: Universal ProbeLibrary probe: # 132. *DNMT*1: 5′- GATGTGGCGTCTGTGAGGT-3′ (forward primer), 5′- CCTTGCAGGCTTTACATTTCC-3′ (reverse primer); Probe: Universal ProbeLibrary probe: # 66. *DNMT3a:* 5′- ACTACATCAGCAAGCGCAAG -3′ (forward primer), 5′- CACAGCATTCATTCCTGCAA-3′ (reverse primer); Probe: Universal ProbeLibrary probe: # 75. *DNMT3b:* 5′- CCGAGAACAAATGGCTTCAG-3′ (forward primer), 5′- TTCCTGCCACAAGACAAACA-3′ (reverse primer); Probe: Universal ProbeLibrary probe: # 64. All assays were designed intron-spanning. The thermal cycler conditions comprised 45 cycles of 95°C for 10 s, 60°C for 30 s, and 72°C for 15 s. Relative mRNA expression was calculated with the Relative Quantification Software (Roche Diagnostics) using an efficiency-corrected algorithm with standard curves and reference gene normalization against *SDHA* and *TBP* (inclusion of a third housekeeping gene (ACTB) led to similar results); These two housekeeping genes have previously been shown to be appropriate for normalization in human glioma and normal brain tissue [23].

### Statistical analysis

The reference point of this study was the date of surgery. Primary endpoint was progression free survival (PFS). Secondary endpoints were overall survival (OS) and treatment response (TR). We assumed the predictive impact of mRNA expression to be at least as high as the impact of the *MGMT* promoter methylation status. Values of MGMT mRNA expression in the biopsy group usually referred to the mean of the expression data obtained from different sites of each tumor. The median of the MGMT mRNA expression of the entire tumor group was used as the cut-off value for definition of the high and the low MGMT mRNA expression group. Based on a previous study of our group [24] we expected a hazard ratio of 0.45 or even less in favor of the group harboring a methylated *MGMT* promoter and/or low MGMT mRNA expression. Accordingly, a sample size in the range of 28 patients in each group was estimated to be sufficient to have a power of 80% to demonstrate a significant difference in PFS in favor of malignant glioma with a methylated *MGMT* promoter and/or low mRNA expression.

PFS and OS were analyzed by the Kaplan-Meier method [25] and compared with the two-sided log-rank test. TR was evaluated after three cycles of TMZ monotherapy according to the McDonald criteria [15]. The Cox model was fitted to asses the prognostic value of the *MGMT* methylation status, MGMT mRNA expression, and other potential prognostic factors. First, the importance of each variable was tested univariately. Forward and backward step-wise proportional hazards modelling was performed to assess the relative and independent prognostic capacity of each parameter. In case of strong interrelationships between covariates, several models were tested and compared with each other (by computing the maximized likelihood). The association between prognostic factors and TR was analyzed with logistic regression models. The distribution of patient- and tumor-related variables between *MGMT* promoter methylated and unmethylated subgroups was analyzed by the chi-squared statistics (for dichotomized variables) and the Wilcoxon test (for continuously scaled variables). In the biopsy group, pair wise comparison of MGMT mRNA data at distant tumor sites was done with the paired T-test. P≤0.05 was considered significant. All calculations were performed using the SAS software package (version 9.2)

Validation of dependency between MGMT mRNA expression and *MGMT* promoter methylation status was performed using data from The Cancer Genome Atlas (TCGA) database (http:tcga.cancer.gov). TCGA glioblastoma samples were supplied by the Broad Institute at the Massachusetts Institute of Technology and the USC Epigenome Center, University of Southern California, USA using the Affymetrix HG-U133A microarray and Illumina Infinium Human DNA Methylation 27 beach chip technology. A total of 209 GBM-samples containing both methylation and gene expression data for the *MGMT* gene was extracted. As level 3 data was used, no additional statistical preprocessing was necessary. The data encompass a total of 20 methylation sites within the *MGMT*-gene. For consistency with our molecular-genetic analyses, only methylation sites were considered that correlated best with gene expression as described by Everhard *et al.* [10] and map to the genomic region covered by the MSP-primers. Beta-values of the remaining methylation sites were averaged for each sample. The median of the beta-values was chosen as the cut-off to classify a sample as being either methylated or non-methylated.

## Results

### Patient characteristics

A total of 63 patients (33 men, 30 women) with a median age of 59 years (range, 25–80 years) were included (Table 1). The median KPS was 70 (range, 60–90). Nineteen patients had deep-seated tumor locations and 30 patients harvested left-sided tumors. Thirty-seven patients underwent molecular stereotactic biopsy procedures (including all patients with AA). Complete tumor resection – as determined by early postoperative MRI – was achieved in 13/26 patients treated with open tumor resection. Histological evaluation revealed a GBM in 53 patients and an AA in 10 patients. All patients were assessable for both determination of the *MGMT* methylation status and MGMT mRNA expression analyses. Treatment included a median number of 6 TMZ cycles for the whole study population, which caused grade 1/2 toxicity in 17 patients and grade 3 toxicity in 2 patients.

**Table 1:** Study population.

		Overall	GBM
**Number of patients**		63	53
**Age**	median	59	62
	range	25–80	25–80
**KPS**	median	70	70
	range	60–90	60–90
**Sex**	female	30	27
	male	33	26
**Tumor side**	right	27	23
	left	30	26
	multifocal	6	4
**Tumor location**	lobar	44	38
	deep-seated	19	15
**Type of surgery**	OP	26	26
	PE	37	27
**Histology**	AA	10	0
	GBM	53	53
**MGMT promoter**	methylated	32	24
	unmethylated	31	29
**MGMT gene expression**	median	0.45	0.50
	range	0.04–1.20	0.07–1.20
	low expression (≤0.45)	32	22
	high expression (>0.45)	31	31
**Treatment**	TMZ cylces (median)	6	5
	range	0–12	0–10
**Progression free survival**	median (month)	10	9
**Overall survival**	median (month)	16	13
**Treatment response** *	tumor control	38	31
	progression	25	22
**Adverse events**	no	44	38
**Adverse events**	yes	19	15

Abbreviations: KPS, Karnofsky performance score; PE, stereotactic biopsy; OP, open tumor resection.

*after 3 months.

### *MGMT* promoter methylation and MGMT mRNA expression

From one single biopsy specimen around 150–800 ng of RNA (260∶280 ratio between 1.8 and 2.1) and 1.5–2 µg DNA were harvested, mainly depending on the size of the individual biopsy specimen. The overall frequency of *MGMT* promoter methylation was 45% (32/63 patients). 8/10 patients with AA and 24/53 patients with a GBM exhibited a methylated *MGMT* promoter (Table 1). The overall median of the MGMT mRNA distribution was 0.45 (range: 0.04–1.2). In thirty-three tumors of the biopsy group at least two samples per tumor (collected from distant sites) were available for both determination of the *MGMT* promoter methylation status and expression analyses (overall number of tissue specimens: 72); the mean distance between the chosen biopsy sites was 9 mm (range 3–38 mm). In the remaining four tumors only one tissue sample was used for molecular-genetic analysis, as the corresponding second ones were suspected to be contaminated by necrotic tissue/blood and/or non-neoplastic tissue (as assumed by the results of both the intraoperative and paraffin embedded analyses of specimens in the direct vicinity of these tissue samples). The *MGMT* promoter methylation profile was homogeneous throughout the viable solid tumor space of those 33 tumors investigated. MSP and bisulfite sequencing exhibited always concordant results. Pairwise comparison of MGMT mRNA expression at different intra-tumoral positions revealed no significant differences (p = 0.79, data not shown).

**Figure 1:**
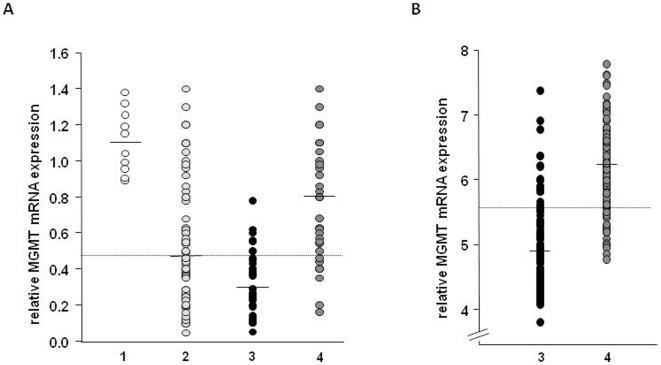
MGMT mRNA expression and MGMT promoter methylation status in malignant glioma. Horizontal bars indicate medians. The dotted line indicates the cut-off value distinguishing low from high MGMT expression values. **A:** Expression of MGMT determined with quantitative real-time PCR in non-cancerous brain tissue specimen (1, N = 10), in malignant glioma (2, N = 63), and in the glioma group stratified by *MGMT* promoter methylation status (methylated, 3, N = 32, and unmethylated, 4, N = 31). cDNA was synthesized from amplified RNA purified from tumor tissue obtained by stereotactic biopsy or open surgery and relative expression of MGMT with respect to expression of the reference genes *SDHA* and *TBP* was determined using real-time PCR. **B:** Validation set obtained from the TCGA database. All data were derived from array analyses and expression levels of methylated (3, N = 105) and unmethylated (4, N = 104) GBM tissue samples are shown.

The median of the low expression group (less equal 0.45) was 0.25, whereas it was 0.8 in the high expression group (>0.45) of the whole study population. Normal brain exhibited the highest expression levels of MGMT mRNA (median: 1.1, p<0.001, Figure 1).

GBM subpopulations that underwent either cytoreductive surgery or stereotactic biopsy did not differ in terms of age, KPS, *MGMT* promoter methylation status, levels of MGMT mRNA expression, applied chemotherapy cycles, and the follow up period. Left sided and/or multifocal tumors were significantly more frequently seen in patients undergoing biopsy only (p<0.01). Patients with AA were significantly younger (median, 55 versus 62 years; p<0.05), showed more frequently a methylated *MGMT* promoter and low expression levels of MGMT mRNA. GBM subpopulations with either a methylated or unmethylated *MGMT* promoter and/or either low or high expression levels of MGMT mRNA did not differ with regard to patients' characteristics. The frequency of *MGMT* promoter methylation and MGMT mRNA expression levels was nearly identical in patients undergoing biopsy only and open tumor resection (data not shown). The degree of MGMT mRNA expression strongly correlated with the *MGMT* promoter methylation status (p<0.0001): The median of the mRNA expression distribution in methylated and unmethylated tumors was 0.26 (range: 0.04–0.78) and 0.8 (range: 0.35–1.2), respectively (Figure 1). Discordant findings were seen in 12 (19%) patients: *MGMT* promoter methylation was associated with high mRNA expression (>0.45) levels in 6 patients (median: 0.58, range 0.46–0.78), whereas low expression levels were seen (≤0.45) in another 6 patients harboring an unmethylated *MGMT* promoter (median: 0.39 range 0.15–0.45). Noteworthy, discordant findings only concerned patients with GBM.

Nearly identical results were obtained by analyzing a publicly available dataset of the TCGA database; 104 out of 209 GBM tissue samples were methylated. The overall median of the MGMT mRNA expression was 5.57 and was congruously used to classify a high and a low expression group. Consistent with our findings, *MGMT* gene expression was strongly associated with methylation status with a median of 4.92 (range 3.79–7.38) for methylated and 6.19 (range 4.77–7.78) for unmethylated samples (p<0.001, Figure 1b). Discordant findings were observed in 46 of 209 samples (22%), which is in accordance to our data (19%). Differences in the range of expression values result from the two different technologies and normalization techniques used (real-time PCR vs. array data).

### DNMTs mRNA expression

**Figure 2:**
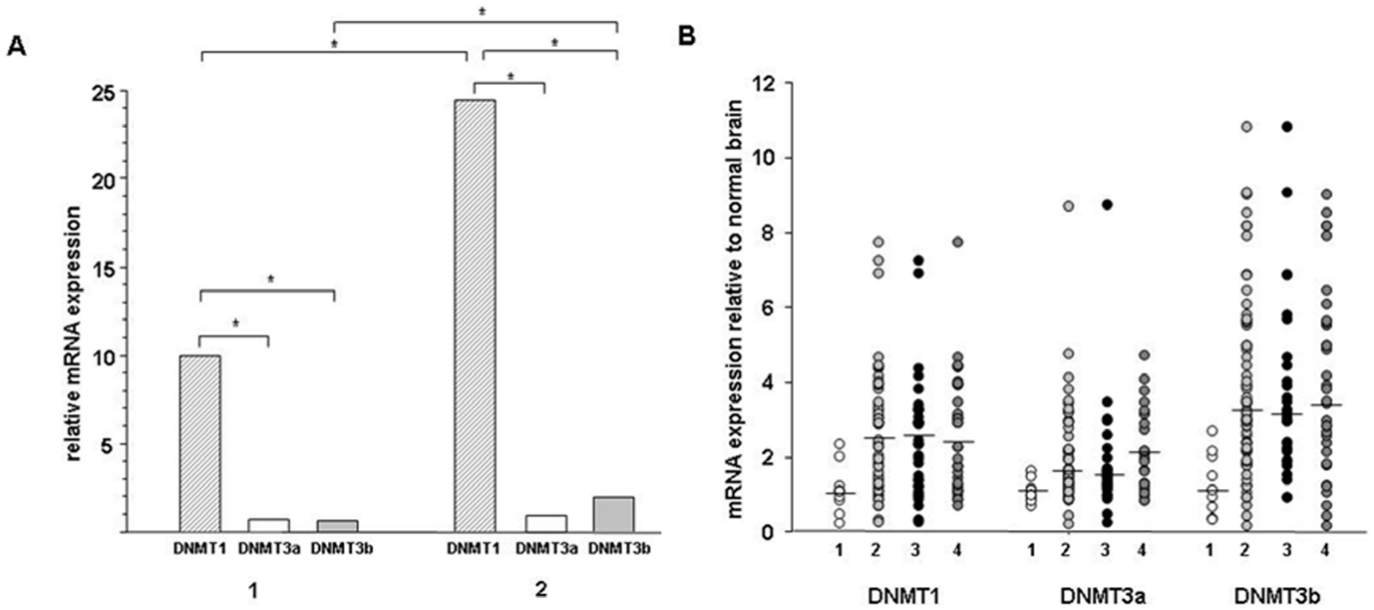
Expression of DNMTs in non-cancerous brain tissue specimen and in malignant glioma. cDNA was synthesized from amplified RNA purified from tumor tissue obtained by stereotactic biopsy or open surgery and expression of DNMTs was determined using real-time PCR. **A:** Expression pattern of DNMTs in normal brain tissue (1, N = 10) and in high grade glioma (2, N = 63). DNMT mRNA expression is calculated relative to the reference genes *SDHA* and *TBP* (*, p>0.01). **B:** Expression of DNMTs in non-cancerous brain tissue specimen (1, N = 10), in high grade glioma (2, N = 63), and in the glioma group stratified by *MGMT* promoter methylation status (methylated, 3, N = 32; unmethylated, 4, N = 31). All data were normalized to the reference genes SDHA and TBP, and the fold change of every DNMT relative to the median expression in the normal brain samples (arbitrarily set to 1) was calculated. Horizontal bars indicate medians.

The mRNA levels of DNMT1, DNMT3a and DNMT3b were analysed in 63 malignant glioma and 10 normal brain samples. Both in normal brain tissue and in tumors DNMT1 was found to be the most expressed methyltransferase (more than 10-fold more expressed than DNMT3a and DNMT3b, Figure 2A). In tumor tissue as compared to normal brain, DNMT1 and DNMT3b were significantly upregulated (DNMT1: 2.5-fold, DNMT3b: 3.2-fold, p<0.001, Figure 2B); the degree of upregulation did not correlate with *MGMT* promoter methylation status and MGMT mRNA expression. For DNMT3a only a trend towards upregulation was detected (1.6-fold, p<0.05); however, the degree of upregulation was more pronounced when stratifying tumors by *MGMT* methylation status: Unmethylated tumors exhibited significant higher DNMT3a mRNA levels than methylated tumors (p = 0.003), and in unmethylated tumors, DNMT3a expression was 2.3-fold increased (p<0.001) as compared to normal brain (Figure 2B). The subgroup analysis of patients with GBM revealed identical results (data not shown).

### Clinical outcome

**Figure 3:**
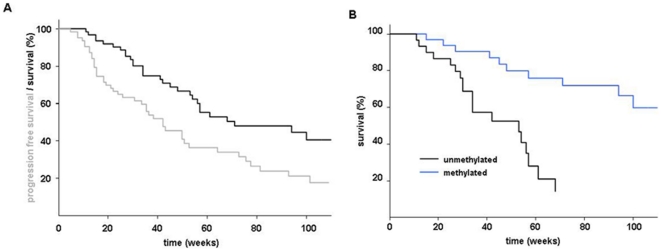
Kaplan-Meier estimates of 63 patients with malignant glioma. Tumor tissue obtained either by stereotactic biopsy or by open surgery. **A:** Progression free survival and overall survival of the whole study population, **B:** Overall survival stratified by the *MGMT* promoter methylation status.

**Figure 4:**
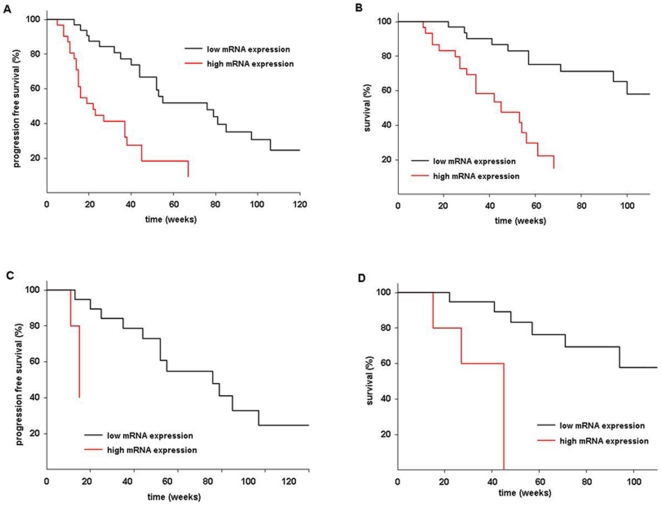
Kaplan-Meier estimates of patients with malignant glioma stratified by MGMT mRNA expression. Tumor tissue obtained either by stereotactic biopsy or by open surgery **A:** Progression free survival (N = 63), **B:** Overall survival (N = 63), **C:** Progression free survival of patients with methylated GBMs (N = 24) **D:** Survival of patients with methylated GBMs (N = 24).

The median follow up time was 10.5 months for the survivors. Forty out of 63 patients exhibited tumor progression and 24 patients died. Death was tumor-related in all patients. No patient was lost to follow up. Fifty-seven patients underwent XRT/TMZ→TMZ treatment and 6 patients with AA primary TMZ chemotherapy, respectively. Kaplan-Meier estimates for PFS and OS of the whole study population are presented in Figure 3A. Treatment response (partial remission or stable disease) was seen in 38/63 patients. Clinical outcome was in favor of *MGMT* promoter methylated tumors and low MGMT mRNA expression: Overall, early treatment response was significantly associated with low MGMT expression (p = 0.004), whereas the influence of *MGMT* promoter methylation was less pronounced (p = 0.02) and even lost for the subgroup of patients with GBM; In the GBM subgroup 19/24 patients with low expression and 12/29 patients with high expression exhibited tumor control or tumor shrinkage three months after XRT/TMZ (p<0.01). Overall, treatment responders experienced a longer OS than non-responders (one year survival rate: 86% vs. 30%, p<0.0001). Promoter methylation correlated with both superior median PFS (18.3 versus 4.9 months) and OS (>22 versus 9.6 months; p<0.0001; Figure 3B). Among patients with a methylated *MGMT* promoter the unadjusted hazard ratio for disease progression and death was 0.22 (95% confidence interval: 0.11–0.46) and 0.2 (95% confidence interval: 0.1–0.47). Stratification for low (≤0.45) vs. high (>0.45) MGMT mRNA expression levels also resulted in a strong correlation with median PFS (17.5 vs. 5 months) and OS (>20 vs. 9.5 months, p<0.0001, Figures 4A, 4B); The unadjusted hazard ratio for disease progression and death was 0.32 (95% confidence interval: 0.14–0.5) and 0.15 (95% confidence interval: 0.06–0.35). Exclusion of anaplastic tumors resulted in nearly identical results concerning the prognostic/predictive impact of both *MGMT* promoter methylation and MGMT mRNA expression (data not shown). In the subgroup of GBM patients with discordant findings stratification for mRNA expression resulted in significant differences for both PFS and OS in case of a methylated *MGMT* promoter: Methylated tumors with high mRNA expression (N = 6) resulted in both shorter PFS (p<0.001, Figure 4C) and OS (p<0.001, Figure 4D) than those with low mRNA expression (N = 18): median PFS and OS was 17.5 months and 21.6 month for the low-expression group, whereas it was 3.3 months and 10.4 months for the high expression group; PFS and OS were similar to that of unmethylated tumors with high MGMT mRNA expression (p>0.3). Conversely, unmethylated GBMs with low mRNA expression (N = 6) did better than those with high mRNA expression (N = 21, data not shown) in term of PFS, and OS; the differences, however, were statistically not significant (p = 0.06); Both PFS and OS was not significantly different to that of methylated tumors with low mRNA expression (p>0.15).

### Prognostic factors

Univariately, *MGMT* promoter methylation (p = 0.0001), low mRNA (p = 0.0004) expression, AA histology (p<0.05) were positively correlated with both increased PFS and OS. Open tumor resection gained prognostic relevance in the subgroup of patients with GBM (p = 0.03). No association was seen between mRNA expression of DNMTs and clinical outcome. Multivariate models including mRNA expression reached a fit as good as those including *MGMT* promoter methylation; it allowed, however, the inclusion of additional variables such as histology and type of surgery (Table 2). The adjusted hazard ratios of *MGMT* promoter methylation and mRNA expression for PFS and OS were consistent with the unadjusted hazard ratios.

**Table 2:** Favorable prognostic factors (uni- and multivariate models).

	Progression Free Survival (PFS)		Overall Survival (OS)	
	p	HR (95% CI)	p	HR (95% CI)
**Univariate**				
*MGMT* methylation	0.0001	0.22 (0.11–0.46)	0.0002	0.2 (0.10–0.47)
Low MGMT mRNA expression	0.0004	0.32 (0.14–0.5)	0.0003	0.15 (0.06–0.35)
Anaplastic astrocytoma	0.02	0.32 (0.12–0.82)	0.03	0.20 (0.05–0.83)
**Multivariate**				
**Model A**				
*MGMT* methylation	0.0001	0.22 (0.11–0.46)	0.0002	0.21 (0.10–0.47)
**Model B**				
Low MGMT mRNA expression	0.0001	0.32 (0.1–0.4)	0.0001	0.12 (0.05–0.31)
Anaplastic astrocytoma	0.004	0.21 (0.11–0.84)	0.009	0.13 (0.03–0.6)
Cytoreductive surgery	0.02	0.45 (0.23–0.88)	0.03	0.33 (0.2–0.83)

## Discussion

Daily clinical practise sometimes indicates discordance between expectations derived from *MGMT* promoter methylation and outcome, and one more recently published study on transcriptional activity in glioblastomas has questioned mechanisms of “direct” transcriptional repression by *MGMT* promoter methylation for a considerable number of tumors: Unmethylated (methylated) tumors were found to express low (high) levels of MGMT mRNA in 15% of the study population [10]. The results of the current study are in line with the findings described by Everhard et *al.* and an additionally performed analysis of an independent validation dataset extracted from the TCGA database. Furthermore, we demonstrate that MGMT mRNA is homogeneously expressed throughout the solid tumor of malignant gliomas, can be reliably determined even from small sized biopsy specimens, is strongly correlated with outcome measurements (even for those with discordant findings), and plays a direct role for mediating tumor sensitivity to alkylating agents. Overall, patients with low MGMT mRNA expression scores did significantly better in terms of TR, PFS, and OS than those with high expression scores. In particular, MGMT mRNA expression retained influence even in those with discordant findings (19% of the series): 6 patients harbouring methylated tumors with high MGMT mRNA expression scores did significantly worse in terms of PFS and OS than their 26 counterparts with concordant findings; outcome was similar to that of unmethylated tumors with high MGMT mRNA expression. A similar pattern was seen in 6 patients with an unmethylated *MGMT* promoter and low MGMT mRNA expression. More data are needed to support the hypothesis that in case of discordant findings expression data could powerfully predict outcome independent of the *MGMT* promoter methylation status.

Concerning the mechanisms underlying the discordant findings, it may be hypothesized that a high MGMT mRNA expression despite a methylated promoter might be due to overruling factors such as increased NF-*k*B activity [26]; low MGMT expression levels combined with an unmethylated promoter might result from transcript destabilization and/or transcription-repressing factors, such as miRNA regulation or histone modifications. However, these issues need to be investigated and in this context, the here described evaluation of MGMT transcriptional activity might be a useful tool. The significant higher MGMT mRNA expression in normal brain (which exhibits an unmethylated *MGMT* promoter) as compared to that of unmethylated tumors also indicates the existence of further mechanisms regulating MGMT expression beyond promoter methylation.

As MSP and bisulfite sequencing do not cover all possible CpG sites of the *MGMT* promoter, it cannot be excluded that omissions of functionally relevant CpG sites may have partly accounted for the detected discrepancies [27]. However, even though some CpG regions have been shown to reflect somewhat better MGMT expression (range of concordant results: 72–85%) in one more recent study [10], no statistically significant difference could be detected for any of the CpG regions investigated: All CpG sites (including those studied by MSP) were highly correlated with MGMT mRNA expression.

As aberrant DNMT expression has been observed in several tumor tissues which might – at least in part – explain epigenetic silencing of selected genes, we estimated the expression of DNMTs in tumor tissue as compared to normal brain, its prognostic/predictive relevance, and its correlation with both the *MGMT* promoter methylation status and MGMT mRNA expression data. In mammals, CGI methylation processes are regulated by DNMT1 (maintenance of DNA methylation pattern) and DNMT3a and DNMT3b (*de novo* methylation) [28]. Aberrant DNMT expression has been observed in various tumor entities relative to normal tissue samples, indicating deregulation of methylation processes in these tumors. For some tumor entities, such as lung carcinoma [29], [30], a correlation between DNMT expression and clinical course was shown. Data describing DNMT mRNA expression in malignant glioma are extremely scarce, indicating an up-regulation of at least DNMT1 and DNMT3b in GBM tissue samples as compared to normal brain [31]. Neither the prognostic/predictive impact of DNMT expression in malignant glioma nor its association with *MGMT* promoter methylation has been analyzed so far. In the current study, we show that in malignant glioma DNMT1 and DNMT3b were significantly upregulated, as compared to normal brain. The degree of upregulation, however, did neither correlate with outcome measurements, nor with *MGMT* promoter methylation status or MGMT mRNA expression. For DNMT3a, only a slight upregulation was detected. Interestingly, unmethylated tumors exhibited significantly higher DNMT3a mRNA levels than methylated tumors. Hence, regulation of the *MGMT* CGI methylation by DNMT3a appears unlikely.

Taken together, the significant upregulation of DNMT1 and DNMT3b indicates their involvement in CGI methylation processes in malignant glioma. However, lack of correlation with clinical outcome makes it reasonable to assume that yet unknown additional mechanisms contribute to the degree of *MGMT* promoter methylation. This aspect certainly deserves further investigation.

### Methodological considerations

We previously showed that MSP and sequence analysis of bisulfite-modified DNA for the determination of the *MGMT* promoter methylation status revealed identical and reproducible results throughout the solid tumor space, even for small amounts of starting DNA as are obtained from a single 1-mm^3^ stereotactic biopsy sample of a malignant glioma [20], [24]. In the current series a previously described new method of combined isolation technique [19] of both RNA and DNA from a single 1-mm^3^ stereotatcatic biopsy sample was used for the first time for MSP, sequence analysis and qPCR. As the extraction of high-quality RNA is the limiting factor in the combined isolation of DNA and RNA, a protocol was used that starts with RNA purification followed by DNA recovery. DNA recovery was approximately 30% reduced compared with routine extraction techniques (i.e. 0.5–1 µg vs 1–1.5 µg from a 1-mm^3^ sample) suggesting that there is a significant DNA loss in the RNA isolation procedure. This loss of DNA, however, appears less relevant for tumors with increased cellularity (such as malignant gliomas). In the current series tissue specimens were snap frozen or processed directly upon withdrawal to guarantee high quality of RNA. qPCR experiments were performed according to the newest MIQE (Minimum Information for Publication of Quantitative Real-Time PCR Experiments) guidelines [32]: All qPCR reactions were efficiency corrected and data were normalized to the geometric mean of two reference genes being determined as suitable for gene expression analyses in human glioma and in glioma compared to normal brain tissue in one of our previous studies [23]. The similar rate of *MGMT* promoter methylation and the similar degree of MGMT mRNA and DNMTs expression in tissue samples obtained from both open tumor resection and molecular stereotactic biopsy technique, and, additionally, the reproducibility of these findings throughout the solid tumor space underscored the validity of the applied methods. It was shown that the applied biopsy technique allows avoiding the contamination of tumor tissue by non-neoplastic tissue in the vast majority of tumors of this series and, more importantly, to recognize contamination if it occurs. It has been reported that lymphocytes, endothelial cells and other types of intra-tumoral non-neoplastic cells such as macrophages/microglias harbouring all an unmethylated *MGMT* promoter might easily bias the determination of both the *MGMT* promoter methylation status and MGMT mRNA expression analysis [33]. The reproducibility of our results throughout the solid tumor space, however, indicates that the impact of these intra-tumoral non-neoplastic cells must be considered minor as compared to that of the solid viable tumor tissue component. However, given the high expression of MGMT mRNA in normal brain the necessity for collecting tissue samples in a highly controlled fashion is underscored [33]. Results concerning the role of MGMT protein expression have been shown to be not conclusive with regard to its correlation with *MGMT* promoter methylation, MGMT mRNA expression, and outcome measurements; inter-observer variability of IHC evaluation, and varying specificity and sensitivity of antibodies might contribute to the observed discrepancies [7]–[9].

Taken together our results show, in accordance to current clinical experience, that *MGMT* promoter methylation status alone does not suffice to provide information about the anticipated clinical course in malignant glioma patients undergoing chemotherapy with alkylating agents. Discordant findings between *MGMT* promoter methylation status and MGMT mRNA expression underscore the necessity to elucidate methylation-independent mechanisms that may regulate MGMT expression.
